# *De Novo* Sequences of *Haloquadratum walsbyi* from Lake Tyrrell, Australia, Reveal a Variable Genomic Landscape

**DOI:** 10.1155/2015/875784

**Published:** 2015-02-01

**Authors:** Benjamin J. Tully, Joanne B. Emerson, Karen Andrade, Jochen J. Brocks, Eric E. Allen, Jillian F. Banfield, Karla B. Heidelberg

**Affiliations:** ^1^Department of Biological Sciences, Dornsife College of Letters, Arts and Sciences, University of Southern California, 3616 Trousdale Parkway, Los Angeles, CA 90089, USA; ^2^Cooperative Institute for Research in Environmental Sciences, CIRES Building, Room 318, University of Colorado Boulder, Boulder, CO 80309, USA; ^3^Department of Environmental Science, Policy and Management, University of California, Berkeley, 54 Mulford Hall, Berkeley, CA 94720, USA; ^4^Research School of Earth Sciences, The Australian National University, Canberra, ACT 0200, Australia; ^5^Division of Biological Sciences, University of California, San Diego, La Jolla, CA 92093-0202, USA; ^6^Marine Biology Research Division, Scripps Institution of Oceanography, La Jolla, CA 92093, USA

## Abstract

Hypersaline systems near salt saturation levels represent an extreme environment, in which organisms grow and survive near the limits of life. One of the abundant members of the microbial communities in hypersaline systems is the square archaeon, *Haloquadratum walsbyi*. Utilizing a short-read metagenome from Lake Tyrrell, a hypersaline ecosystem in Victoria, Australia, we performed a comparative genomic analysis of *H. walsbyi* to better understand the extent of variation between strains/subspecies. Results revealed that previously isolated strains/subspecies do not fully describe the complete repertoire of the genomic landscape present in *H. walsbyi*. Rearrangements, insertions, and deletions were observed for the Lake Tyrrell derived *Haloquadratum* genomes and were supported by environmental *de novo* sequences, including shifts in the dominant genomic landscape of the two most abundant strains. Analysis pertaining to halomucins indicated that homologs for this large protein are not a feature common for all species of *Haloquadratum*. Further, we analyzed ATP-binding cassette transporters (ABC-type transporters) for evidence of niche partitioning between different strains/subspecies. We were able to identify unique and variable transporter subunits from all five genomes analyzed and the *de novo* environmental sequences, suggesting that differences in nutrient and carbon source acquisition may play a role in maintaining distinct strains/subspecies.

## 1. Background

The microbiology of low diversity, extreme hypersaline environments has been extensively studied providing a more detailed understanding of extant communities [1–4]. The microorganisms, including predominantly halophilic Archaea, in these extreme environments thrive in high salt concentrations that define that which can sustain life, providing experimental model systems to evaluate the limits of microbial growth. Biological stressors in these environments are not limited to high salt concentrations but also include high temperatures, intense UV exposure, fluctuating oxygen levels, and variable ionic ratios that can impact the establishment of electrochemical gradients [5]. Even with limiting oxygen concentrations, many of the microorganisms in these systems grow optimally under aerobic, heterotrophic conditions [6].

*H. walsbyi* is known for its dominance in hypersaline environments and its unique square-shaped morphology [7].* H. walsbyi* is an obligate halophile and has a number of adaptations that appear linking its ability to grow in environments with high salt concentrations. Due to its flat cell structure,* H. walsbyi* has the highest surface area-to-volume (s/v) ratio of any microbe. Individual cells can measure 2 *μ*m × 2 *μ*m × 0.2 *μ*m, but sheets of cells have been shown to grow on scales up to 20–40 *μ*m^2^ [8]. The immense size of the* H. walsbyi* sheets is possible as the s/v ratio is only dependent on the thickness of the cells. This high s/v ratio is directly linked to the importance of membrane processes in cell development [9].* H. walsbyi* has large suite of cellular transport proteins essential to maintain its predominantly heterotrophic lifestyle [10]. However, it is also capable of utilizing bacteriorhodopsins to support phototrophic growth [11]. This process is enhanced by the presence of bacteriorhodopsin proteins on both faces of the cell, as incident light can pass through the thin cellular cytoplasm [10].* H. walsbyi* is nonmotile but utilizes gas vesicles to position itself within the water column to presumably maximize both irradiance [10] and available oxygen, as the solubility of oxygen in hypersaline environments is low [12] and the removal of oxygen through heterotrophic processes results in the limited penetration of oxygen within shallow hypersaline systems.* H. walsbyi* is unique amongst the class Halobacteriaceae in that it has a substantially lower genomic percent G + C (%G + C) content [13–15]. Two hypotheses have been proposed to explain this sharp deviation from the other halophilic Archaea. First, low %G + C is thought to be common among marine oligotrophs as one possible mechanism for reducing nitrogen demands [16]. More likely the low %G + C helps to decrease DNA stability caused by the overstabilization of high internal Mg^2+^ concentrations, in a similar fashion as to how thermophiles have increased %G + C to increase DNA stability in high temperature environments [17]. Interestingly, 16S rRNA gene studies have shown that* H. walsbyi* has limited divergence (≤2%) in the 16S rRNA gene [4] on a global scale compared to other groups within the Halobacteriaceae (~7%), indicating that* H. walsbyi* may be more evolutionarily constrained.

To date, the most extensive genomic comparison of* H. walsbyi* has focused on two isolated strains cultured from salterns in Spain (str. DSM16790) [9] and Australia (str. C23) [18] that have been fully sequenced [10, 19]. Some previous metagenomic work was performed from the Spain saltern using fosmid end clone libraries to explore* H. walsbyi* diversity within the system and revealed that there was underlying diversity within the system, including novel* Haloquadratum*-related clones and the identification of four genomic islands [13, 20]. Comparative analysis of the complete DSM16790 and C23 genomes revealed a lack of genomic rearrangements with much of the variation between the genomes occurring as the result of insertions/deletions of small- and large-scale genomic regions [19]. The limited genomic variation of these two genomes and low 16S rRNA gene divergence has led to a proposed hypothesis that* H. walsbyi* is globally homogenous in genomic content and is either highly dispersed or under strict evolutionary constraints, resulting in limited biogeography [19]. Expanding comparative analysis of* Haloquadratum* genomes beyond previous results identified from fosmid-based metagenomes [13, 20] would allow for a better understanding of conserved genomic properties within the genus.

Previous results from Lake Tyrrell, Victoria, Australia, a naturally occurring thalassohaline hypersaline lake, have been used to reconstruct several genomes for uncultured [21] and novel strains of halophilic Archaea [14, 22] and viruses [23, 24]. Previous work on halophilic Archaea in this system was performed on metagenomic samples collected in 2007 and 2008 using long-read Sanger- and 454-derived sequences. Several draft and composite genomes were generated using the long-read Sanger dataset from 2007 Lake Tyrrell metagenome, resulting in two additional* H. walsbyi* genomes (J07HQW1 and J07HQW2), as well as the first genome of a separate candidate species of the genus* Haloquadratum* (J07HQX50) [14]. In this study, we use metagenomic samples collected in 2010 using short-read Illumina paired-end sequences to explore the genomic diversity of the most abundant halophilic Archaea in the system,* Haloquadratum walsbyi*. We used* Haloquadratum*-related assemblies of the 2010 Lake Tyrrell metagenome to specifically evaluate* H. walsbyi* heterogeneity. Comparative analysis of the five* Haloquadratum* genomes and the environmental assemblies has revealed new insights into the genomic landscape of these abundant halophilic Archaea.

## 2. Materials and Methods

### 2.1. Sample Collection and Metagenome Sequencing

Samples were collected using a serial filtration method in January 2010 (Austral summer) from evaporative brine samples collected over a 4-day time-series experiment at Lake Tyrrell in Western Victoria, Australia (35.52°S, 142,80°W). As has been reported in Heidelberg et al. [25], salt concentrations were typically > 300 g L^−1^, and samples were collected from a shallow (<20 cm depth) pool separated from the main lake by a salt barrier. As described in Narasingarao et al. [21] and Podell et al. [14], the water samples were prefiltered through a 20 *μ*m Nytex screen and then serially filtered through 3.0, 0.8, and 0.1 *μ*m polyethersulfone filters (Pall Corporation, NY, USA). DNA was extracted from the 0.1 *μ*m, 0.8 *μ*m, and 3.0 *μ*m filters, as described in Heidelberg et al. [25]. Sequencing was performed at the J. Craig Venter Institute (JCVI, Rockville, MD, USA) using a paired 2 × 100 bp Illumina sequencing platform with a 100 bp insert size.

### 2.2. Assembly, Binning, and Annotation

Samples were assembled using an iterative process of assembly using IDBA-UD (v. 1.1.0) [26], binning via hierarchical clustering of tetranucleotide sequences, and sequence recruitment using the BWA aligner (v. 0.6.1-r104) [27] and the Ref_Select function of the program suite, SEAStAR (v. 0.4.17) [28]. For all assemblies, IDBA-UD was used with the default settings and the precorrection setting (- -pre_correction) turned on. For all read recruitment steps, the FASTQ files for the forward and reverse reads were aligned to the contiguous DNA sequences (contigs) using the BWA aligner with the* aln* program to generate the alignment and* samse* to generate the corresponding SAM file, with the maximum number of differences allowed for both processes set at four (-n 4). FASTA files were then generated using the ref_select command within SEAStAR.

For the first round of assembly, genomic data from each sample filter was independently assembled to generate contigs. Contigs greater than 5,000 bp in length were binned using hierarchical clustering of tetranucleotide sequences using Pearson's correlation value of 0.80 as a cutoff. The putative coding DNA sequences (CDSs) for all clusters of contigs with a total span of greater than 10,000 bp were determined using the program FragGeneScan (v. 1.16) [29]. Translated putative CDSs were compared using BLAST against a database of the protein sequences of 16 organisms from the family Halobacteriaceae, two organisms from the class Nanohaloarchaea (see Supplemental Table S1 available online at http://dx.doi.org/10.1155/2014/875784). The contigs from all bins for which >60% of the translated putative CDS were assigned to the genus* Haloquadratum* were retained and used to recruit the corresponding sequence reads using SEAStAR.

For the second round of assembly, multiple filters from the same sample were assembled together. Contigs greater than 5,000 bp in length were then clustered, as above. Based on assessment of the clusters, it was determined that a lower threshold of Pearson's correlation (0.50) would sufficiently capture similar contigs. Thirteen bins were identified with a total span of contigs > 1 Mb (largest bin = 6.4 Mb). The sequence reads from each individual filter were then recruited to the contigs within each of the identified bins (e.g., sample LT71 was divided into three bins containing greater than 1 Mb of sequence. The sequences from the 0.1 *μ*m and 0.8 *μ*m filters were then recruited against the contigs of each bin separately to generate new FASTA files).

For the final round of assembly, sequences from each bin and the corresponding filter were assembled (e.g., sequences from the 0.1 *μ*m filter of sample LT71 would be in 3 FASTA files representing the bins identified in the second round of assembly). For further analysis of the* de novo* assemblies, only contigs > 50,000 bp in length were considered. Putative CDSs were determined using the RAST Annotation Server [30] with the following settings applied: gene caller = RAST; release64; automatically fix errors = yes; backfill gaps = yes.

### 2.3. Recruitment Statistics

The genome sequences of the five currently available* Haloquadratum* species were downloaded from the IMG database (*Haloquadratum walsbyi* C23, DSM16790, J07HQW1, J07HQW2, and* Haloquadratum* sp. J07HQX50) [31]. The genomes of J07HQW1, J07HQW2, and J07HQX50 were reconstructed from environmental samples collected in 2007 from Lake Tyrrell. The J07HQW1 and J07HQW2 genomes have high coverage with long-read Sanger sequencing (9.0X and 8.7X, resp.) and limited gaps along the length of a single closed scaffold (gaps along 0.2% and 0.5% of the total predicted genome, resp.), for which all of the gaps are shorter in length (~50–100 bp) than is spanned by both the plasmids (8–10 kbp) and fosmids (35 kbp) used to generate sequence reads. All genomes were used in conjunction with the binned contigs generated after the second round of assembly to recruit, using SEAStAR, the maximum number of putative* Haloquadratum*-related sequences from all of the samples and filters. This pool of redundant sequences included those recruited from the entire Lake Tyrrell metagenome against the IMG genomes and pooled sequence reads from each filter of each sample against the set of contigs derived after the second round of assembly. The recruited sequences were processed to remove duplicated sequences and then identical sequences using the unique.seqs command from the mothur suite of bioinformatic tools [32]. The sequences were then processed to remove sequences that had become unmated during the process. The remaining pool of sequences was aligned against the* Haloquadratum* genomes, including extrachromosomal DNA. Alignments were performed using Geneious (v. 6.1.6) using the “Map to Reference” program with the following settings applied: medium-low sensitivity/fast; iterate up to 5 times; do not trim.

The three samples that had different filter fraction sizes (LT71, LT80, and LT85) were aligned to the* Haloquadratum* genomes derived previously from Lake Tyrrell (J07HQW1, J07HQW2, and J07HQX50) to estimate the relative abundance of each organism in different filter fractions. Alignments to the genomes were performed as shown above.

### 2.4. Whole Genome Alignments and Genome Synteny

Contigs greater than 50,000 bp in length generated after the third round of assembly were aligned to the J07HQW1 genome using the progressive MAUVE genome aligner [33]. Contigs were manually assigned into one of four categories based on the degree of synteny compared to the reference genome: high, medium, low, and no synteny. Contigs were assigned to the high category of synteny if the resulting alignment indicated that the entire contig contained colinearized regions compared to J07HQW1 or approximately 70–90% of the contig had colinearized regions, but these regions were rearranged compared to J07HQW1. Contigs were assigned to medium category if approximately 30–70% of the contigs had colinearized regions. The low category contained sequences with less than 30% of the contig possessed colinearized regions. The none category had little to no alignment to the reference genome. Contigs in the no synteny category were further aligned to the other* Haloquadratum* genomes to determine if these sequences represented regions in the other organisms but not contained in the J07HQW1 genome.

Large regions of the J07HQW1 genome to which many environmental contigs aligned were identified for further analysis. The contigs and the corresponding segment of the J07HQW1 genome were aligned using the progressive MAUVE genome aligner to the* Haloquadratum walsbyi* genomes (C23, DSM16790, and J07HQW2) and manually inspected for insertions, deletions, and rearrangements. Putative gene content for all insertions and deletions was determined where possible.

### 2.5. Genes of Interest: Halomucins and ABC-Type Transporters

Halomucins were annotated previously in the* Haloquadratum walsbyi* C23 and DSM16790 genomes. These sequences were used to identify similar sequences in the previously assembled genomes from Lake Tyrrell and the environmental contigs generated for this research. Alignments of putative CDSs were performed using the CLUSTALW [34] with the following settings applied: cost matrix = IUB; gap open cost = 15; gap extend cost = 6.66. Putative signal peptides were identified using SignalP (v. 4.1) with cutoffs between 0.45 and 0.51 [35].

A database was constructed from all of the putative protein sequences related to ABC-type transporters, including all subunits of the transporters (i.e., ATPase, substrate-binding, and permease), from all of the previously annotated* Haloquadratum* genomes. Each* Haloquadratum* genome was then compared using BLAST [36] to a subset of this database that did not include proteins within that specific genome. ABC-type transporters were identified from the environmental annotations and compared to the total database using BLAST. BLAST results were parsed for ABC-type transporter subunits that did not have significant BLAST matches to the database or had less than 80% AAID. Putative substrates and functions of the subunits were derived from available annotations and not independently verified.

## 3. Results and Discussion

### 3.1. Sample Collection and Metagenome Sequencing

Surface water from five time points was sampled via filtration over the course of 4 days from Lake Tyrrell in January 2010. Based on library construction and sequencing success, eight samples were chosen for inclusion in this study, including three time points for which both small (0.1 *μ*m) and large (0.8 or 3.0 *μ*m) filter fractions were obtained. The final metagenome consisted of 135,239,438 trimmed, high quality, and paired-end sequences that contained 12.7 Gbp of data (Table 1).

### 3.2. Assembly and Binning

An iterative assembly and binning process was used to reduce complexity and enrich* Haloquadratum* sequences in the combined dataset. The initial round of assembly generated 5,403 contigs greater than 5,000 bp in length, for which 856 bins were generated using hierarchical clustering of tetranucleotide frequencies. Of these 856 bins, 424 were putative* Haloquadratum* origin based on comparison to a database of class Halobacteriaceae and class Nanohaloarchaea, containing 2,096 contigs. Sequence reads were recruited to the contigs putatively related to* Haloquadratum*. The second round of assembly resulted in a total of 1,965 of the generated contigs that were >5,000 bp in length. These contigs were subjected to tetranucleotide hierarchical clustering, as above; however, visual inspection of the clustering relationship suggested that Pearson's correlation cutoff of 0.50 would be more inclusive of the assembly results (i.e., generating larger bins), while simultaneously dividing the dataset into distinct genomic units (Supplemental Figure S1). In total, 13 bins contained over 1 Mbp in assemblies, with the largest bin containing 6.4 Mbp of sequence data.

For the final round of assembly, the sequence reads from each filter fraction were recruited against the contigs within each bin from a single sample and reassembled (i.e., sample LT71 had 2 filter fractions and 3 identified bins; each filter was recruited against each bin, such that 6 total assemblies were performed) (Supplemental Table S2). Results from this round of assembly indicated that, for assembly statistics, including N50, mean length, and total length, the values increased. Only the maximum length statistic had a relatively small decline, but this decrease was offset by the increase in both N50 and mean length (Table 2; Supplemental Table S2). The third round of assembly produced 195 contigs at greater than 50,000 bp in length, which was used for further analysis. Annotations of the contigs identified 27,801 putative CDSs (for additional results see Supplemental Information).

### 3.3. Recruitment to Reference Genomes

Several of the 2010 Lake Tyrrell metagenome samples had multiple sequenced filter fractions (LT71, LT80, and LT85), which included a small filter fraction (0.1 *μ*m) and either a 0.8 *μ*m filter (LT71) or a 3.0 *μ*m filter (LT80 and LT82). Recruiting the 2010 metagenomic sequences against the genomes assembled from the 2007 Lake Tyrrell metagenome revealed the genomes recruited 13–30% of the total library from the 3.0 *μ*m filter, compared to <7% of the library from the 0.1 *μ*m filters. These results suggest that a majority of the* Haloquadratum* populations in the Lake Tyrrell system exist as aggregates larger than 3.0 *μ*m in size and expand on results identified in a 16S rDNA analysis of the Spanish saltern from which DSM16790 was isolated [13] (for additional results see Supplemental Information).

A comparative analysis of two* H. walsbyi* genomes from cultured representative originally isolated from salterns in Spain and SE Australia has suggested that* H. walsbyi*, as a species, is mostly uniform in genomic content across a large dispersal range implying a lack of biogeography for these organisms [19]. Heterogeneity within the Spanish saltern had been observed previously in an end-sequenced fosmid metagenome [13]. Sequencing of full-length fosmids revealed genomic islands (GIs) along the DSM16790 genome and determined that about ~50% of fully sequenced fosmids were nonsyntenic [20]. However, the number of available* H. walsbyi* genomes and the depth of sequencing limited both fosmid metagenome analyses. The Lake Tyrrell genomes were derived from environmental sequences from a portion of the lake used for commercial salt production, similar to saltern crystallizer ponds, while previously genomes of* Haloquadratum* were obtained using laboratory isolates [10, 19]. The Lake Tyrrell genomes and* H. walsbyi* C23 are both derived from hypersaline environments in Australia separated by ~330 km allowing for further testing of the uniform nature of their genomic content. Recruitment of the environmental sequences against the* Haloquadratum* genomes, including DSM16790 and C23, was used to determine how related the Lake Tyrrell environmental sequences were to the finished genomes.

A subset of 128,992,632 redundant sequences was recruited to the* Haloquadratum* genomes and the contigs generated after the second round of assembly. Redundant sequences were removed via a two-step procedure: removal of (1) copies of the same sequences that were recruited to multiple reference genomes and (2) sequences that were identical. Unpaired sequences were removed, resulting in 20,477,772 nonredundant, paired, and unique sequences.

Results from the alignment of these sequences indicate that the most completely covered genome by the environmental sequences was the J07HQW1 genome (99.96% coverage) followed closely by the J07HQW2 genome (99.7% coverage) (Table 3). The coverage of J07HQW1 and J07HQW2 is higher than the coverage of the C23 (92.3% coverage) and DSM16790 (93.3% coverage) genomes and substantially higher than the J07HQX50 genome (mean = 84.0% coverage). The lower coverage of the J07HQX50 genome is expected, as previous results have shown that J07HQX50 represents a smaller proportion of the* Haloquadratum* population (6.9% compared to 74.1% in 2007), and therefore the nonredundant sequences will contain substantially less J07HQX50-like sequences resulting in lower coverage of the genome. The mean coverage depth for the* H. walsbyi* genomes is greater than 222 times coverage (range = 0–6,090X coverage). The C23 and DSM16790 genomes have similar values for mean coverage to J07HQW1 and J07HQW2 but do not have the same percent coverage, suggesting that there is a distinction between the genomic contents of these* Haloquadratum *species. Coverage of the PL6B plasmid suggests it may be present in upwards of 32–40% of the* Haloquadratum* population (see Supplemental Information). Both J07HQW1 and J07HQW2 were not completely aligned across 100% of their respective genomes, potentially indicating that there has also been some genomic variation in the form of insertions or deletions that has occurred in approximately three years since the genomes were sampled, though there is the potential for stochastic variation in the shotgun sequencing methodology.

### 3.4. Whole Genome Alignments

To explore the extent to which genome content and rearrangements influenced the alignment results for the* H. walsbyi* genomes, whole genome alignments were performed for the genomes and the environmental contigs generated after the third round of assembly. The 195 contigs > 50,000 bp in length were aligned to the J07HQW1 genome. The contigs in the high and medium synteny category, 82 and 53, respectively, had successful alignments covering much of the reference genome. For the 32 contigs assigned to the no synteny category, it was determined that all of the contigs could be assigned as related to either J07HQW2 or J07HQX50 based on alignments to the respective genome. While some of these contigs contained segments that possessed novel gene content (data not shown), a majority of the contigs, for all of the categories, could be identified as having genomic architecture similar to one of the three Lake Tyrrell* Haloquadratum* genomes.

Regions were selected that highlight large-scale genomic rearrangements and heterogeneous gene content. From the initial alignments that spanned the length of the J07HQW1 genome, 14 regions were identified along the J07HQW1 genome that met the criteria for further examination; the region had to (1) be at least 60,000 bp in length and (2) have multiple environmental contigs spanning the region of interest. 122 contigs were identified from these regions of interest. For each region of interest, the corresponding environmental contigs and* H. walsbyi* genomes were aligned together. These data revealed numerous features previously unseen in the comparison with C23 and DSM16790, including large genomic rearrangements and substantial genomic insertions and deletions. Three such variable regions are presented in detail below (additional information for each region in Supplemental Information).

#### 3.4.1. Region Spanning 600,000–770,000 bp along J07HQW1

**Figure 1:**
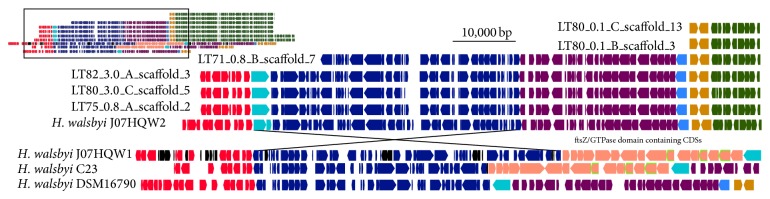
Inset: a gene map of the region spanning 600,000–770,000 bp along the* H. walsbyi* J07HQW1 genome, the corresponding regions in the other* H. walsbyi* genomes, and the environmental contigs. Full image: an expanded view of the region denoted in the inset. Syntenic regions are marked by matching colors. Pink arrows denote the 16 CDS segment identified as unique to the* H. walsbyi* J07HQW1 and C23 genomes. Environmental contigs use a long form identification number whereby the first item is the sample (e.g., LT71), the second item is the filter fraction (e.g., 0.8 *μ*m), the third item is the phylogenetic bin (e.g., B), and the fourth item is the scaffold number (e.g., scaffold 13). The regions represented on the gene map from each genome are as follows: J07HQW1, 610,420–769,897 bp; J07HQW2, 2,470,010–2,313,700 bp; C23, 510,000–630,000 bp; DSM16790, 510,000–610,000 bp.

This region spans ~170 kbp of the J07HQW1 genome, but the corresponding regions in the other* H. walsbyi* genomes are smaller in scale as a result of a large insertion/deletion of 16 CDSs common for J07HQW1 and C23, plus additional 33 insertions along the J07HQW1 genome (Figure 1). Many of the 33 insertions along the J07HQW1 genome appear to be noncoding, although there were several annotated transposase or transposase-like CDSs, as well as annotated CDSs with putative cellular functions. The 16 CDS segment of J07HQW1 and C23 is poorly annotated but contains several homologs of* ftsZ*/GTPase domain containing CDSs, a gene family required for successful cell division, specifically in the formation of daughter cells.

There are five environmental contigs that appear to be more closely related to the J07HQW2 genome due to the lack of the 16 CDS segment, described above, and the presence of a ~50 kbp inversion in the same genomic landscape near the insertion segment found in C23 and J07HQW1. The full length of the environmental contigs is syntenic to the J07HQW2 genome. Interestingly, DSM16790 lacks both the 16 CDS segment and the inversion seen in J07HQW2 and the environmental contigs, suggesting that there are at least three potential orientations for this segment, and the inserted/deleted sequences are not required for the inversion. This result is interesting because numerous results indicated that J07HQW1 and J07HQW2 are present in the environment in about equal abundance. However, for the region all six environmental contigs (from four different samples) possess the J07HQW2 orientation. While difficult to understand completely, as this result may be due to the incomplete nature of metagenomic sampling, this could be evidence of a change in the dominant genomic architecture for this genomic region in Lake Tyrrell to the J07HQW2 orientation.

#### 3.4.2. Region Spanning 1,600,000–1,660,000 bp along J07HQW1

**Figure 2:**
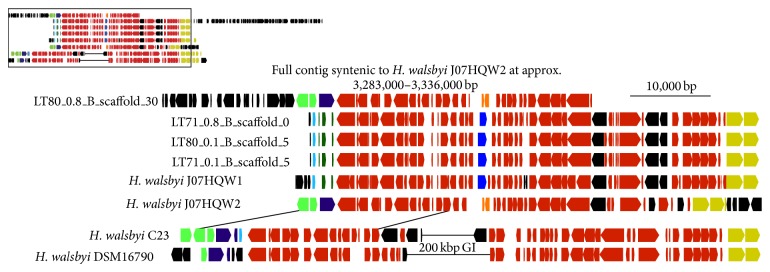
Inset: a gene map of the region spanning 1,600,000–1,660,000 bp along the* H. walsbyi* J07HQW1 genome, the corresponding regions in the other* H. walsbyi genomes*, and the environmental contigs. Full image: an expanded view of the region denoted in the inset. Syntenic regions are marked by matching colors. Environmental contigs use a long form identification number (see Figure 1 caption). The regions represented on the gene map from each genome are as follows: J07HQW1, 1,600,465–1,660,398 bp; J07HQW2, 3,299,582–3,359,276 bp; C23, 1,240,000–1,270,000 bp and 1,530,000–1,490,000 bp; DSM16790, 1,240,690–1,270,000 bp and 1,510,000–1,470,000 bp.

This region is shared between J07HQW1 and J07HQW2 but is split over two portions of the C23 and DSM16790 genomes separated by ~200 kbp (approximate positions: 1,240–1,270 kbp and 1,470–1,530 kbp), though all four genomes have similar gene content (Figure 2). The ~200 kbp region present in C23 and DSM16790 has previously been identified as a genomic island [20]. Gene content variation between the* H. walsbyi* genomes includes a number of putative CDSs with predicted functions.

The four environmental contigs have a high degree of similarity between J07HQW1 and J07HQW2, but the gene content suggests that for this region the dominant genomic architecture is that of J07HQW1. The longest environmental contig (ID: LT71_0.8_B_scaffold_0) has an additional ~40 kbp segment at the end of the sequence compared to the other contigs. This segment is a large rearrangement relative to J07HQW1 and is syntenic to a segment of the genome at the approximate position, 2,232–2,284 kbp. A single ~55 kbp environmental contig (ID: LT80_0.1_B_scaffold_30) has full synteny to the J07HQW2 genome (approximate position 3,283–3,336 kbp). Unlike the above region, these contigs support previous research that suggests the J07HQW1 genomic architecture is the more abundant gene synteny in the Lake Tyrrell system, while J07HQW2 represents a second distinct synteny. Yet despite the similarities, the largest environmental contig still represents a large-scale rearrangement of the J07HQW1 genome, potentially suggesting a genomic landscape undergoing episodes of rearrangement.

#### 3.4.3. Region Spanning 2,619,000–2,702,000 bp along J07HQW1

**Figure 3:**
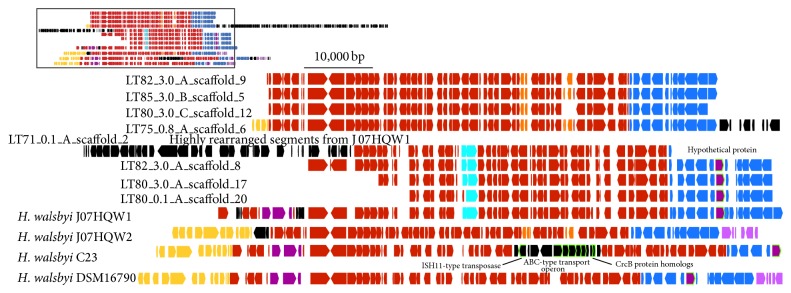
Inset: a gene map of the region spanning 2,619,000–2,702,000 bp along the* H. walsbyi* J07HQW1 genome, the corresponding regions in the other* H. walsbyi genomes*, and the environmental contigs. Full image: an expanded view of the region denoted in the inset. Syntenic regions are marked by matching colors. Arrows highlighted in green denote individual sequences specifically identified in the paper. Environmental contigs use a long form identification number (see Figure 1 caption). The regions represented on the gene map from each genome are as follows: J07HQW1, 2,619,269–2,703,165 bp; J07HQW2, 1,229,374–1,314,534 bp; C23, 2,110,000–2,220,000 bp; DSM16790, 2,030,000–2,125,000 bp.

For this region of interest, the overall genomic structure is conserved for all four* H. walsbyi* genomes (Figure 3). There are several gene indels, including a defining feature of J07HQW1, C23, and DSM16790 compared to J07HQW2, in the form of a hypothetical protein (J07HQW1_02778). C23 has an insertion of 13 CDSs that includes a number of annotated genes, such as an ISH11-type transposase (Hqrw_3137), an ABC-type transport operon (ATPase, substrate-binding, and membrane permease subunits) without an annotated target substrate (Hqrw_3141-3145), and two homologs of CrcB proteins (Hqrw_3147 and 3148), related to camphor resistance and chromosome condensation.

The environmental contigs can be separated into those related to J07HQW1 (3 contigs) and those related to J07HQW2 (4 contigs) based on the presence/absence of the hypothetical protein mentioned above. The longest of the four contigs (ID: LT75_0.8_A_scaffold_6) (~160 kbp) is fully syntenic to the J07HQW2 genome (1,240–1,400 kbp). An ~86 kbp contig (ID: LT71_0.1_A_scaffold_2) contains a high degree of rearrangement compared to the J07HQW1 genome along a ~40 kbp span of the contig. This span is syntenic to seven different segments of the J07HQW1 genome and while there is some synteny to the other* Haloquadratum* genomes all of the other alignments have substantial differences between the sequences. Unlike the two previously discussed regions, the split between J07HQW1- and J07HQW2-like contigs is closer to the predicted abundances of these two species in the environment, though LT71_0.1_A_scaffold_2 has a unique genomic structure that has not been previously seen, representing a novel orientation of the* H. walsbyi* genome.

The* de novo* environmental contigs for the regions described above overwhelming can be assigned to one of the three* Haloquadratum* reference genomes generated from the 2007 Lake Tyrrell metagenome. Interestingly, despite evidence suggesting that J07HQW1 and J07HQW2 represent equal proportions of the* Haloquadratum* population, two of the three regions had aligned contigs that indicate one of the two* H. walsbyi* strains was the dominant genomic landscape in the environment. The* de novo* contigs do not accurately represent abundance in the environment but offer an estimate of presence/absence of each region among environmental strains. While stochastic processes may influence the set of* de novo* contigs aligned to the reference genomes, the reproduction of similar contigs with the same gene synteny from multiple samples and filters suggests that the results may represent an accurate snapshot of the genomic landscape of the* Haloquadratum* community and demonstrate recombination events that have swept to a majority of the* H. walsbyi* individuals. Further, much of the observed variability is between the genomes of the* H. walsbyi* strains; only a minority of the* de novo* contigs demonstrate genomic variation and rearrangement, suggesting that the majority of the* H. walsbyi* individuals within the community represent stable gene synteny that has persisted over the three-year period since J07HQW1 and J07HQW2 were sampled.

### 3.5. Genes of Interest

#### 3.5.1. Halomucins

In the C23 and DSM16790 genomes, several large protein-coding sequences (>7,000 amino acids) were identified as mucin homologs, a family of high molecular weight proteins that prevent desiccation, and were given the term halomucin (*hmu*). These proteins are proposed to have a role in protecting the cell against desiccation and/or creating a localized environment with higher water activity than the surrounding environment [10, 19]. Both of the J07HQW1 and J07HQW2 genomes lack annotated* hmu* genes or putative candidates.

The only indication of a fully functional* hmu* homolog for sequences generated from Lake Tyrrell appears to be a putative CDS on an environmental contig (referred to as* ehmu1*; short-hand for environmental-*hmu*; ID: LT85_0.1_A_scaffold_0).* ehmu1* is the longest putative CDS annotation for the 195 environmental contigs. It is similar in length to the C23 annotated Hmu (7,243 AA versus 7,836 AA, resp.), although both are smaller than the DSM16790 annotated HmuI (9,159 AA). The Hmu homologs of DSM16790 and C23 share 73.8% amino acid identity (AAID), while eHmu1 has 46.0% and 47.9% AAID to DSM16790 and C23, respectively. eHmu1 has several regions in the alignments to DSM16790 and C23 that have > 75% AAID, suggesting that several of the domains common to the Hmu protein are present in eHmu1. Signal peptide prediction of eHmu1 suggests that this putative CDS would undergo translocation across the membrane. Collectively, this evidence suggests that eHmu1 is a homolog of the* hmu* gene.

**Figure 4:**
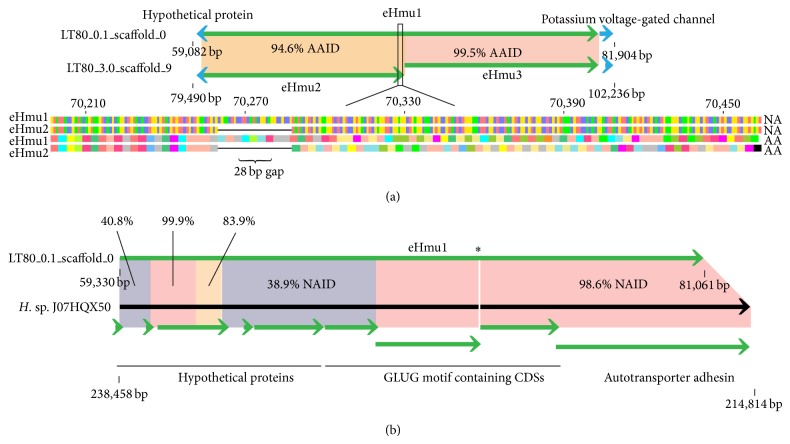
(a) A comparison of halomucin homologs, eHmu1, found on the environmental contig LT80_0.1_scaffold_0 and the corresponding fragments of eHmu2 and eHmu3, found on the contig LT80_3.0_scaffold_9. The section indicated in the black box reveals the nature of the 28 bp deletion within eHmu2 that causes the frameshift resulting in a stop codon approx. 230 bp downstream. AAID: amino acid identity. (b) A comparison of the halomucin homolog eHmu1 and the corresponding region and annotations identified in* H. *sp. J07HQX50. ∗ represents the location of an 81 bp gap in a GLUG motif containing CDS from* H. *sp. J07HQX50. NAID: nucleic acid identity.

The second and third longest putative CDSs had identical annotations (“large exoprotein involved in heme utilization or adhesion”), were adjacent to each other on a contig from the same sample as eHmu1, but from a different filter fraction (ID: LT85_3.0_A_scaffold_9), and collectively had a length similar to eHmu1 (3,701 AA + 3,527 AA = 7,218 AA). One of these putative CDS could be aligned to the C-terminus of eHmu1 with 99.5% AAID (eHmu2) and the other to the N-terminus of eHmu1 with 94.6% AAID (eHmu3). eHmu2 lacked a detectable signal peptide sequence, while eHmu3 possessed an N-terminus signal peptide, suggesting that it could be translocated across the membrane. Examination of the alignment of eHmu2 and eHmu3 against eHmu1 reveals two deletions relative to the structure of eHmu1, with a 28 bp deletion resulting in a frameshift/stop codon (Figure 4(a)). For many bacteria and Archaea, a stop codon in a putative CDS will generally result in a nonfunctional product. The nature of eHmu2 and eHmu3 implicates that these putative CDSs represent a partially degraded environmental Hmu homolog.

Evidence for another degraded* hmu* homolog occurs in the J07HQX50 genome. The longest putative CDS in the genome is 2,413 AA and is annotated as “autotransporter adhesin.” The protein sequence of this putative CDS has 99.7% AAID to the C-terminus of eHmu1. Sequences upstream of the “autotransporter adhesin” contain five more putative CDSs with a collective length of 7,129 AA (2,413 + 989 + 1,285 + 667 + 878 + 897 AA). One of these CDSs (“hypothetical protein,” 878 AA) contains an N-terminus signal peptide sequence. A nucleotide alignment of this segment of the J07QX50 genome (214,814–238,458 bp) reveals that there are areas that have high nucleic acid identity (NAID) with the* ehmu1* sequence with two different portions having >98.0% NAID, while the adjacent portions had 83.9%, 40.8%, and 38.9% NAID, as well as an 81 bp gap (Figure 4(b)). The data suggest this series of putative CDSs represents a highly degraded* hmu*-like sequence. For the three* hmu* homologs detected in the dataset, two appear to be degraded to the point of no longer being functional. eHmu1 appears to be a complete* hmu* homolog with the necessary signal peptides required for translocation across the membrane and may play a role by increasing the water activity near the cell in a high salt, low water activity environment. The lack of evidence for a* hmu* homolog in the J07HQW1 and J07HQW2 genomes and the degraded nature of eHmu2, eHmu3, and the putative CDSs of the J07HQX50 genome indicates that functional* hmu* genes are not a universal adaptation for the genus [19] and suggests that other factors may be driving the successful adaption of* Haloquadratum* in high salt environments. However, it is possible that, due to the role plasmids play in haloarchaeal communities, halomucins are present on extrachromosomal genetic elements, but there is currently only evidence to support halomucins as a feature of the genomic chromosome.

#### 3.5.2. ABC-Type Transport Systems

One particular area of interest in microbial ecology is how microorganisms/strains can successfully occupy the same physical environment without evolutionary pressures driving an extinction event. Evidence suggests that J07HQW1 and J07HQW2 have persisted as distinct strains and J07HQX50 as a different species, over at least three years, such that it is likely that each occupies noncompetitive ecological niches. In examining these niche differentiations, special attention was paid to putative CDSs with annotations related to ATP-binding cassette (ABC) type transporters, a system of active transport that requires the use of ATP to move substrates across the cell membrane. These transport proteins were specifically targeted for this research because they represent the substrates for which organisms are expending energy to move across the membrane. Transport protein activity could govern the available niches that each species may occupy. Additionally, transporter protein variation can be a form of adaptation against viral predation, preventing attachment of viral particles to cell membrane.

The annotated ABC-type transporters for each individual genome and the putative CDSs annotated from the environmental contigs were compared to a database of the genome transporters. The goal was to identify ABC-type transporter components that either (1) do not have an ortholog in the other organisms or (2) have a degree of divergence relative to other genomes. A cutoff of 80% AAID was used to determine if a transporter sequence was divergent from those present in the other* Haloquadratum* species. This divergence is particularly relevant for the substrate-binding and permease subunits of the transporters, as changes in the protein structure may impact the specificity and efficiency of substrate transport. Variation in the ATPase subunit is likely to have a limited impact, as the functional aspect must remain consistent for the transporter to function.

The* Haloquadratum* genomes had between 138 and 159 annotated ABC-type transport subunits. In general, comparisons between the genomes only revealed a few protein sequences that fell below the 80% AAID threshold. For C23 and DSM16790, the average similarity for transporter components above the threshold was ~98-99% AAID. For the Lake Tyrrell genomes, the average similarity of components above the threshold was ~89–93% AAID. These values are expected as J07HQW2 (91% AAID) and J07HQX50 (89% AAID) are more divergent organisms within the* Haloquadratum* clade compared to J07HQW1, C23, and DSM16790. The high average similarity of transporter subunits above the 80% AAID threshold suggests that the diversity below the threshold represents subunits with potentially variable transport properties.

None of the* H. walsbyi* strains contained unique ABC-type transporter components. However, each genome did have a distinct set of variant transport components that indicate differentiations between the species/strains (Supplemental Table S3). The results from C23 revealed 10 of 150 ABC-type transporter subunits below the 80% AAID threshold. Of the 10, there were two copper (Cu) permeases (Hqrw_4112 and Hqrw_1178) and zinc (Zn) (Hqrw_2414) and urea/short-chain amides substrate-binding (Hqrw_4030) subunits. In addition to this, there was a full operonic ABC-type transporter without an assigned function (Hqrw_3142-3145). In comparison, DSM16790 had eight variant subunits (of 146 annotated subunits); five came from a complete operon related to branched-chain amino acids (HQ2192A-HQ2197A). J07HQW2 also has subunits related to branched-chain amino acid transport, but it does not include the full operon, only the permease (J07HQW2_03665 and 03668) and ATPase (J07HQW2_03669 and 03670). The other three variant subunits from DSM16790 are related to lipoprotein transport (two permeases and an ATPase) (HQ3476A-3478A), indicating that, unlike C23, which appears to have only variant transporters related to metallic ions and amides transport, DSM16790 may have variant specificity/efficiency in transporting lipoproteins.

J07HQW1 had 11 variant transporter subunits of 141 annotated subunits. Five of the 11 are ATPases, two are annotated as “hypothetical proteins” (J07HQW1_00367 and 00669), and the remaining four are two antimicrobial peptide (J07HQW1_00014 and 00042), one nucleoside (J07HQW1_01905), and one Fe^3+^-hydroxamate (J07HQW1_00956) permeases. The Fe^3+^-hydroxamate permease has binding sites for both Fe and cobalamin, both of which are competitively scavenged in the Lake Tyrrell system. The antimicrobial peptide permeases could be indicative of allelopathy between J07HQW1 and other organisms within the system.

J07HQW2 had the largest suite of variant transporter subunits for the* H. walsbyi* species with 29 of 159 subunits determined to be variants, 13 of which were annotated as ATPases (Supplemental Table S3). J07HQW2 has variant permease and substrate-binding subunits for nitrate/sulfonate/bicarbonate, spermidine/putrescine, di-/oligopeptide/Ni, phosphate/phosphonate, branched-chain amino acids, and phosphate (PhoT family transporters). The heterotrophic nature of these transport subunits is expected for* Haloquadratum*. These transporters are present in the other* H. walsbyi* genomes, but the variant substrate-binding and permease subunits of J07HQW2 may possibly indicate different sources/specificities of the substrates for these transporters.

Similar to J07HQW2, J07HQX50 has an entire suite of variant transporters with similar annotations to those found in the other* Haloquadratum* genomes, but these differences may be indicative of the niche differentiation between the genera (Supplemental Table S3). J07HQX50 has 138 annotated ABC-type transporter subunits. Three of these subunits were identified as not having an ortholog in the other genomes. One annotation (J07HQXv2_01450) had no similarity to other sequences in the GenBank nonredundant database and was <150 bp in length. The other two annotated subunits without* Haloquadratum* orthologs include a spermidine/putrescine substrate-binding protein (J07HQXv2_01469) and a glycine betaine/choline-binding lipoprotein permease (J07HQXv2_02756). J07HQX50 has 86 variant transporter subunits many of whose predicted substrates have been observed in the variants of the other genomes, including spermidine/putrescine, phosphate (PhoT and PstA family transporters), di-/oligopeptides, Fe^3+^, cobalamin, amides, cobalt, and branched-chain amino acids. Interestingly, 30 of the variant transporter subunits are annotated as sugar, carbohydrate, and/or monosaccharide subunits. The variety of sugar-related transporters in J07HQX50 may be an indication of the ecological niche that this organism occupies in the system, utilizing a specific subset of simple sugars that allow it to remain present in the system along with the more dominant species.

The environmental contigs had 734 ABC-type transporter subunits identifiable based on RAST annotations. This number includes some degree of redundancy, as there were many contigs within the dataset that may represent the same organism but collected on a different filter fraction or in a different sample. Specific numbers for any individual transporter subunit are not considered as evidence of abundance or importance. As such, five subunits were identified on the environmental contigs that did not have orthologs in the* Haloquadratum* genomes. These subunits included two unassigned ATPases, urea and nitrate substrate-binding proteins, and a nonfunctional fragment of an oligopeptide transport protein. The environmental contigs contained 96 variant transporter subunits (Supplemental Table S3). As with J07HQW2 and J07HQX50, many of the predicted substrates for these subunits are related to the heterotrophic nature of these organisms. One group of variant transporter subunits that was not identified in the other* Haloquadratum* genomes was predicted to utilize glycerol-3-phosphate and dihydroxyacetone (DHA) as substrates. It has been shown that DSM16790 has the genetic potential to convert both DHA and glycerol-3-phosphate into dihydroxyacetone phosphate (DHAP) [10]. DHAP has been shown to be an important intermediate within the phosphate and carbon metabolisms for DSM16790 because it can be used as substrate for gluconeogenesis or glycolysis or converted via glycerol-1-phosphate into a key component of archaeal membrane lipids [37]. The presence of variants for these transport units within the environmental contigs and not in the analysis of the* Haloquadratum* genomes indicates that alternative forms of these subunits may be contributing to niche maintenance in the Lake Tyrrell system.

## 4. Concluding Remarks

Detailed genomic comparisons between our environmental metagenome and the publically available* Haloquadratum* genomes have revealed a number of new aspects of how these organisms thrive in hypersaline environments. Specifically, this research indicates that the previous assumptions made regarding the homogenous nature of* H. walsbyi* gene synteny are not supported when comparisons are made between more representatives of the species and genus. We found both small-scale insertions/deletions in genomic content (i.e., single genes, e.g., transport proteins and hydrolases) and large-scale rearrangements and variation in genomic content. Further, previous results suggesting that halomucins play a crucial role in the adaptation of* Haloquadratum* to high salt environments are not supported by results from Lake Tyrrell. While some small portion of Lake Tyrrell* Haloquadratum* populations has an intact halomucin homolog, there is evidence that this particular gene is under negative selective pressure in both the environment and J07HQX50. As such, halomucins probably do not represent the only mechanism by which* Haloquadratum* spp. maintain cellular homeostasis but are part of a suite of potential mechanisms that allow for growth in high salt environments. Lastly, analysis of ABC-type transporters has given insight into how each member of the genus* Haloquadratum* may maintain niche differentiation in the same physical environment, including variations in transporters for metal cofactors (e.g., Fe, Cu, Ni, and Co), phosphonates/phosphates, amides (e.g., spermidine and putrescine), and carbohydrates (e.g., monosaccharides).

## Supplementary Material

The Supplementary Material: provides increased levels of analysis and discussion in regards to the “Assembly and Binning”, recruitment of the metagenome on to the *H. walsbyi* genomes and plasmids from different filter fractions, and the whole genome alignment segments presented in the manuscript, including an additional fourth region of alignment spanning 3,033,000-3,387,000 bp along J07HQW1.
Supplementary figures: illustrate the raw results of the tetranucleotide binning protocol and offer more details for the whole genome aligned segments. Supplementary tables include details regarding the *Halobacteriaceae* genomes used to construct the local database, changes to assembly efficiency through the various round of assembly, the genes identified as variant ABC-transporter subunits, and percent recruitment of the Lake Tyrrell *H. walsbyi* genomes from different filter fractions of the metagenome.

## Figures and Tables

**Table 1:** 

Sample	Date	Time (24 hr)	Temperature (°C) [11]	TDS (wt%)^a^ [11]	pH [11]	Filter size	Number of trimmed sequences in library
LT71 (site 1)	Jan. 7, 2010	07:45	20	32	7.2	0.1	12,845,524
	Jan. 7, 2011	07:45	20	32	7.2	0.8	4,357,176
LT75 (site 1)	Jan. 7, 2010	20:00	32	36	7.3	0.8	13,475,176
LT80 (site 1)	Jan. 9, 2010	16:50	45	27	7.1	0.1	20,609,138
	Jan. 9, 2011	16:50	45	27	7.1	3.0	9,014,584
LT82 (site 1)	Jan. 10, 2010	12:50	33	32	7.2	3.0	15,358,950
LT85 (site 2)	Jan. 10, 2010	12:50	37	35	7.1	0.1	52,520,328
	Jan. 10, 2011	12:50	37	35	7.1	3.0	7,058,560

^a^Total dissolved solids, weight percent.

Data from Temp (°C), TDS, and pH were original published in Emerson et al. 2012 [23].

**Table 2:** 

Assembly comparison	Mean ratio of third round to second round assemblies	Standard deviation	Median
N50	1.28	0.48	1.20
Maximum Contig Length	0.76	0.24	0.74
Mean Contig Length	1.03	0.47	0.90
Total Length Contained in Contigs	1.10	0.20	1.16

**Table 3:** 

Genome ID	Source	Maximum coverage	Mean coverage	% Coverage	Number of recruited sequences (10^6^)
*H. walsbyi *J07HQW1	Metagenome Lake Tyrrell, AUS (2007) [8]	2075	275.1	99.96	10.58
*H. walsbyi *J07HQW2	Metagenome Lake Tyrrell, AUS (2007) [8]	6090	222.3	99.7	8.78
*H. *sp. J07HQX50 Scaffold 1	Metagenome Lake Tyrrell, AUS (2007) [8]	2101	97.6	84.6	1.68
*H. *sp. J07HQX50 Scaffold 2	Metagenome Lake Tyrrell, AUS (2007) [8]	2064	98	83.4	1.6
*H. walsbyi *C23	Cultured isolate from Geelong, AUS [23]	3387	254.2	92.3	8.754
*H. walsbyi *C23 Plasmid PL6A	Cultured isolate from Geelong, AUS [23]	240	45.4	98.2	0.00332
*H. walsbyi *C23 Plasmid PL6B	Cultured isolate from Geelong, AUS [23]	484	90.1	98.6	0.006635
*H. walsbyi *C23 Plasmid PL100	Cultured isolate from Geelong, AUS [23]	1016	80.1	95.9	0.086
*H. walsbyi *DSM16790	Cultured isolate from Alicante, Spain [15]	3368	254.9	93.3	8.76
*H. walsbyi *DSM16790 Plasmid	Cultured isolate from Alicante, Spain [15]	515	66.1	71.5	0.034
