# Supplementary material for: De Novo Sequences of Haloquadratum walsbyi from Lake Tyrrell, Australia, Reveal a Variable Genomic Landscape
Source: Archaea. 2015 Feb 1;2015:875784. doi: 10.1155/2015/875784 (PMC4330952; doi:10.1155/2015/875784)
Supplement: Supplementary file 1 — The Supplementary Material: provides increased levels of analysis and discussion in regards to the “Assembly and Binning”, recruitment of the metagenome on to the H. walsbyi genomes and plasmids from different filter fractions, and the whole genome alignment segments presented in the manuscript, including an additional fourth region of alignment spanning 3,033,000-3,387,000 bp along J07HQW1. Supplementary figures: illustrate the raw results of the tetranucleotide binning protocol and offer more details for the whole genome aligned segments. Supplementary tables include details regarding the Halobacteriaceae genomes used to construct the local database, changes to assembly efficiency through the various round of assembly, the genes identified as variant ABC-transporter subunits, and percent recruitment of the Lake Tyrrell H. walsbyi genomes from different filter fractions of the metagenome. [file 875784.f1.pdf]

Supplemental Information

Assembly and binning

An iterative assembly and binning process was used to reduce complexity and enrich for *Haloquadratum* sequences in the combined dataset. The initial round of assembly generated 5,403 contigs greater than 5,000 bp in length, for which 856 bins were generated using hierarchical clustering of tetranucleotide frequencies. Of these 856 bins, 424 were determined to be of putative *Haloquadratum* origin, containing 2,096 contigs. A database of reference genomes containing representatives of Class *Halobacteriaceae* and Class *Nanohaloarchaea* (Phylum Nanohaloarchaeota; [1]) was generated from the IMG genome database [2]. *Haloquadratum* is a distinct phylogenetic group within the *Halobacteria* [3, 4] and this genomic signature resulted in a sharp distinction between bins above 60% assignable *Haloquadratum*-like putative CDSs versus those well below 50% (data not shown). The proportion of contigs determined to be *Haloquadratum*-like represented a nearly identical proportion of the total number of contigs as found in a previous metagenomic study of microbial populations in Lake Tyrrell in 2007 (38.8%) [5]. This result indicates that the first round of assembly and binning captured a portion of the total metagenomic dataset that is similar to the previous relative abundance of *Haloquadratum*. These contigs provided an ideal situation for generating more refined genomic constructs.

Sequence reads were recruited to the contigs putatively related to *Haloquadratum*. This reduced the number of sequence reads undergoing the second round of assembly by between 62 and 93% (Mean = 79%). Samples with multiple filter fractions had both filter fractions assembled together in an effort to include sequences that may bridge gaps in the previous assembly. The second round of assembly resulted in a reduction in the total number of contigs generated and an increase in the N50 and mean length of the contigs (Supplemental Table S2). These results were expected, as targeting the *Haloquadratum* portion of the metagenome decreases the total number of genomic assemblies than can be generated and decreases the likelihood of assembly breakpoints in highly conserved regions. A total of 1,965 of the generated contigs were greater than 5,000 bp in length. These contigs were subjected to tetranucleotide hierarchical clustering, as above, however, visual inspection of the clustering relationship suggested that a Pearson's correlation cutoff of 0.50 would be more inclusive of the assembly results (*i.e.*, generating larger bins), while simultaneously dividing the dataset into distinct genomic units (Supplemental Figure S1). The inclusive nature of the bins was determined to be

acceptable for two reasons: (1) comparisons were made between bins and not within bins; and (2) the third round of assembly results would examine only sequences greater than 50,000 bp in length, such that poorly assembling subgroups within the bin would not be included in the final results. In total, 13 bins contained over 1 Mbp in assemblies, with the largest bin containing 6.4 Mbp of sequence data.

For the final round of assembly, the sequence reads from each filter fraction were recruited against the contigs within each bin from a single sample and re-assembled (*i.e.*, sample LT71 had 2 filter fractions and 3 identified bins; each filter was recruited against each bin, such that 6 total assemblies were performed) (Supplemental Table S2). Results from this round of assembly indicated that for assembly statistics, including N50, Mean Length, and Total Length, the values increased. Only the Maximum Length statistic had a relatively small decline, but this decrease was offset by the increase in both N50 and Mean Length. Simultaneously, the third round of assembly allowed for the separation of distinct populations via tetranucleotide binning and captured any potential differences between organisms captured on different filters (Table 2; Supplemental Table S2). The third round of assembly produced 195 contigs at greater than 50,000 bp in length, which was used for further analysis. Annotations of the contigs identified 27,801 putative CDSs.

Recruitment to reference genomes

<u>Recruitment variations between filters</u>

Several of the samples collected in 2010 for this study had multiple filters sequenced in an effort to capture a wider spectrum of organisms, including the genus *Dunaliella*, a species of green microalgae that have been shown to be the dominant primary producer in other hypersaline environments [6]. The larger filter fractions were sequenced to capture the genomic potential of this organism to determine its role in the Lake Tyrrell system, but further offered an opportunity to understand *Haloquadratum* in the environment. The samples with multiple filter fractions (LT71, LT80, and LT85) include the smallest filter fraction (0.1 μm) and either a 0.8 μm filter (LT71) or a 3.0 μm filter (LT80 and LT82). The three *Haloquadratum* genomes (*H. walsbyi* J07HQW1 and J07HQW2, and *H.* sp. J07HQX50) generated from the 2007 Lake Tyrrell metagenome were used to recruit environmental sequences from the different filter fractions of this study to determine which fraction contained the most *Haloquadratum*-related sequences.

The previous Lake Tyrrell metagenome was constructed only utilizing sequences from the 0.8 μm and 0.1 μm filters. In that study, about 38% of the assembled microbial populations were assigned to *Haloquadratum* [5, 7]. However, *Haloquadratum* can grow in several different morphotypes, as single, square cells (~2 μm^2) or as sheets of cells (~12-40 μm^2) [26]. Results from the recruitment of the three different filter fractions used in this study (0.1, 0.8, and 3.0 μm), indicated that for each of the three *Haloquadratum* genomes the larger of the two filters recruited more sequences than the 0.1 μm filters. The genomes recruit between 13-30% of the total library from the 3.0 μm filter, compared to less than 7% of the library from the 0.1 μm filters (Supplemental Table S4). These results suggest that a majority of the *Haloquadratum* populations in the Lake Tyrrell system exist as aggregates larger than 3.0 μm in size and expand on results identified in a 16S rDNA analysis of the Spanish saltern from which DSM16790 was isolated [6]. As such, previous estimates that examined data from the 0.8 and 0.1 μm filters to determine the relative abundance of *Haloquadratum* in Lake Tyrrell may be an underestimation, and further sequencing of the 20 μm size fraction may reveal more *Haloquadratum* diversity. Further, results suggested that J07HQW1 was more representative of the environment on both size filters in the LT71 and LT80 samples, but this trend was weakened/reversed in the LT85 sample, while J07HQX50 is substantially less abundant. These results are expected as the previous Lake Tyrrell studies have indicated near identical abundances of J07HQW1 and J07HQW2 and lower abundances of J07HQX50.

Recruitment variations between *Haloquadratum* plasmids

In previous research [8], particular interest has been paid to the presence of the extrachromosomal DNA related to *H. walsbyi* in the form of plasmids. These plasmid sequences were included during the recruitment and alignment processes to elucidate the degree to which they may be represented in the environmental data. Plasmid PL6A and PL6B were shown to have similarity to sequences derived from the 2007 Lake Tyrrell metagenome samples [8]. The results from the 2010 metagenome indicate that the PL6A and PL6B plasmids have a percent coverage similar to that of J07HQW1 and J0HQW2, while the other identified plasmids (PL100 and DSM16790 plasmid) were recruited less highly (Table 4). These results suggest that some variation of the identified plasmids is present in the population, but that gene content differences may account for gaps in coverage. Further, it is possible to get a sense for how widely distributed these plasmids are in the Lake Tyrrell populations. If every cell possessed a copy of the plasmid,

the mean coverage value for the genomes and the plasmid should be similar. Results show that the PL6B plasmid has the highest mean coverage and, using mean coverage of the genomes as a value to indicate abundance, PL6B is present in upwards 32-40% of the *Haloquadratum* population (assuming a single copy per cell). The other plasmids have lower mean coverage and, therefore, likely present in a smaller proportion of the population.

Whole genome alignments

Region spanning 600,000-770,000 bp along J07HQW1

This region spans ~170 kbp of the J07HQW1 genome, but the corresponding regions in the other *H. walsbyi* genomes are smaller in scale (~80-120 kbp) as a result of a large insertion/deletion of 16 CDSs common for J07HQW1 and C23, plus an additional 33 insertions along the J07HQW1 genome (Supplemental Figure S2). Many of the 33 insertion along the J07HQW1 genome appear to be non-coding, although there are five annotated transposase or transposase-like CDSs (J07HQW1_00711, 00712, 00744, 00751, and 00752), an annotated amino glycoside phosphotransferase (J07HQW1_00662), which can confer resistance to some amino glycoside antibiotic compounds, and a Kef-type potassium (K^+) transporter (J07HQW1_00654). The 16 CDS segment of J07HQW1 and C23 is poorly annotated, but contains several homologs of *ftsZ*/GTPase domain containing CDSs (J07HQW1_00735, 00739, and 00741), a gene family required for successful cell division, specifically in the formation of daughter cells.

There are five environmental contigs that appear to be more closely related to the J07HQW2 genome due to the lack of the 16 CDS segment, described above, and the presence of a ~50 kbp inversion in the same genomic landscape near the insertion segment found in C23 and J07HQW1. Interestingly, DSM16790 lacks both the 16 CDS segment and the inversion seen in J07HQW2 and the environmental contigs, suggesting that there are at least three potential orientations for this segment, and the inserted/deleted sequences are not required for the inversion.

Further supporting the relationship between the environmental contigs and J07HQW2 is the nature of the downstream portion of the environmental contigs. Using the longest contig as a reference (ID: LT75_0.8_A_scaffold_2), the first ~80 kbp is syntenic with the J07HQW1 genome, though inverted, while the latter ~75 kbp is syntenic to a different portion of the

genome, spanning from 250-340 kbp along the J07HQW1 genome. However, the full length of the environmental contigs is syntenic to the J07HQW2 genome. This result is interesting because the results from previous assessment of Lake Tyrrell and from this study of *Haloquadratum* populations indicates that J07HQW1 and J07HQW2 are present in the environment in about equal abundance, with J07HQW1 being slightly more abundant. However, for the region all six environmental contigs (from four different samples) only possess the J07HQW2 orientation. While difficult to understand completely, as this result may be due to the incomplete nature of metagenomic sampling, this could be evidence of a change in the dominant genomic architecture for this region in the system to the J07HQW2 orientation.

Region spanning 1,600,000-1,660,000 bp along J07HQW1

This region is shared between J07HQW1 and J07HQW2, but is split over two portions of the C23 and DSM16790 genomes separated by ~200 kbp (Approx. positions: 1,240-1,270 kbp and 1,470-1,530 kbp), though all four genomes have similar gene content (Supplemental Figure S3). The ~200 kbp region only present in C23 and DSM16790 has previously been identified as a genomic island [9]. There are several notable exceptions to the shared gene content, including a transposon that has inserted within a phosphoesterase (Hqrw_2209 and 2210) in the C23 genome and an ISH9-type transposon (HQ2030A) insertion adjacent to a pterin-4-alpha carbinolamine dehydratase (HQ2029A), involved in phenylalanine hydroxylation, in the DSM16790 genome. Downstream of the ISH9-type transposon insertion there is a conserved intergenic sequence present in C23 and J07HQW2, indicating that this insertion may be a recent acquisition and/or that the non-coding sequence is under selection preventing genetic variation. Further, gene content variation between the *H. walsbyi* genomes includes a metal-dependent hydrolase (J07HQW1_01677) present in J07HQW1 and J07HQW2, an esterase/lipase (J07HQW1_01684) and predicted flavoprotein involved in K^+ transport (J07HQW1_01683) in J07HQW1, and two uncharacterized iron-sulfur domain proteins (J07HQW2_03631 and 03634) in J07HQW2.

The four environmental contigs have a high degree of similarity between J07HQW1 and J07HQW2, but the gene content suggests that for this region the dominant genomic architecture is that of J07HQW1. The longest environmental contig (ID: LT71_0.8_B_scaffold_0) has an additional ~40 kbp segment at the end of the sequence compared to the other contigs. This segment is a large rearrangement relative to J07HQW1 and is syntenic to a segment of the

genome at the approximate position, 2,232-2,284 kbp. A single ~55 kbp environmental contig (ID: LT80_0.1_B_scaffold_30) has full synteny to the J07HQW2 genome in a region from approximately 3,283-3,336 kbp. Unlike the above region, these contigs support previous research that suggests the J07HQW1 genomic architecture is the more abundant gene structure in the Lake Tyrrell system, while J07HQW2 represents a second distinct structure. Yet despite the similarities, the largest environmental contig still represents a large-scale rearrangement of the J07HQW1 genome, potentially suggesting a genomic landscape undergoing episodes of rearrangement.

Region spanning 2,619,000-2,702,000 bp along J07HQW1

For this region of interest, the overall genomic structure is conserved for all four *H. walsbyi* genomes (Supplemental Figure S4). There are several exceptions, including a defining feature of J07HQW1, C23, and DSM16790 compared to J07HQW2, in the form of a hypothetical protein (J07HQW1_02778). J07HQW1 has a number of smaller insertions, relative to the other sequences, most of which are intergenic. Annotated differences include an IS605 family transposase (J07HQW1_02712) and two CDSs related to amino acid transport (J07HQW1_02743 and 02744). C23 has an insertion of 13 CDSs that includes a number of annotated genes, such as a ISH11-type transposase (Hqrw_3137), an ABC-type transport operon (ATPase, substrate-binding, and membrane permease subunits) without an annotated target substrate (Hqrw_3141-3145), and two homologs of a CrcB proteins (Hqrw_3147 and 3148), related to camphor resistance and chromosome condensation.

The environmental contigs can be separated into those related to J07HQW1 (3 contigs) and those related to J07HQW2 (4 contigs) based on the presence/absence of the hypothetical protein mentioned above. The longest of the four contigs (ID: LT75_0.8_A_scaffold_6) (~160 kbp) is fully syntenic to the J07HQW2 genome (1,240-1,400 kbp). This environmental contig and the J07HQW2 genome are represented in this region and a second region spanning 2,715-2,820 kbp along the J07HQW1 genome. An ~86 kbp contig (ID: LT71_0.1_A_scaffold_2) contains a high degree of rearrangement compared to the J07HQW1 genome along a ~40 kbp span of the contig. This span is syntenic to seven different segments of the J07HQW1 genome and while there is some synteny to the other *Haloquadratum* genomes, all of the other alignments have substantial differences between the sequences. Unlike the two previously

discussed regions, the split between J07HQW1- and J07HQW2-like contigs is closer to the predicted abundances of these two species in the environment, though LT71_0.1_A_scaffold_2 has a unique genomic structure that has not been seen previously, representing a novel orientation of the *H. walsbyi* genome.

<u>Region spanning 3,033,000-3,387,000 bp along J07HQW1</u>

Comparatively, this region and the relationship between the *H. walsbyi* genomes is the most complex of the regions discussed above. The genomic structure of this region splits into two different orientations based on a large rearrangement and two inversions, one orientation for the Lake Tyrrell genomes and one for C23 and DSM16790 (Supplemental Figure S5). Along with the changes in the genomic orientation, each of the genomes has variations in the gene content. J07HQW1 has 26 small variations relative to the other genomes, but many of these differences occur within intergenic spaces. Some of the large gaps involved annotated genes with functions such as deoxycytidine deaminase (J07HQW1_03374), involved in pyrimidine salvaging, archaeosine tRNA-ribosyltransferase (J07HQW1_03421), involved in production of the nucleotide archaeosine, and an IS605 family transposase (J07HQW1_03431). The J07HQW2 genome has two insertions relative to the other genomes that include an IS605 family transposase (J07HQW2_01798) and the subunits of an ABC-type phosphonate transporter (J07HQW2_01803-5). Both C23 and DSM16790 are well conserved in relation to each other, except for two large insertions in each of the genomes. The C23 genome contains a 13 CDSs segment predominantly composed of hypothetical proteins, but includes a ISH10-type transposase (Hqrw_3737), a signal transducing histidine kinase (Hqrw_3735), and CPxCG-related small zinc finger protein (Hqrw_3740), which been shown to regulate bacteriorhodopsin in *Halobacterium salinarum* [29]. The DSM16790 genome contains a 29 CDSs segment that putatively appears to be a phage insertion. Along with 17 hypothetical proteins, this segment includes an annotated phage integrase (HQ3271A), a probable type II restriction enzyme (HQ3275A), an ATP-dependent helicase (HQ3276A), and a bacterial conjugation protein homolog (HQ3291A). Collectively, these results reveal a region of the *H. walsbyi* genomes that is highly variable and is continually targeted for gene insertions/deletions.

There are 12 environmental contigs that align to different portions of the region in J07HQW1. These contigs can be divided, based on distinction in gene content, into putative

groups related to J07HQW1 (3 contigs) and J07HQW2 (4 contigs), as well as a third group (4 contigs) related to *H.* sp. J07HQX50, the third dominant *Haloquadratum* species in the Lake Tyrrell system, and a fourth group with a single environmental contig which had a unique genomic structure (ID: LT80_0.1_A_scaffold_3). The key differentiation between the J07HQW1-related contigs and the J07HQW2-related contigs is the presence of the phosphonate transport operon mentioned above. The longest contig (ID: LT75_0.8_A_scaffold_0) related to J07HQW2 additionally possesses a 6.2 kbp insertion that includes a putative exported protein, an annotated tricarboxylate membrane transport protein, and two hypothetical proteins, compared to the *H. walsbyi* genomes. The contigs related to J07HQX50 have a similar gene synteny compared to the *H. walsbyi* genomes. One feature of J07HQX50 and the longest related contig (ID: LT75_0.8_B_scaffold_0) is an ~20 kbp putative phage insertion site. Along with the annotated phage integrase are putative CDSs annotated as the components for an ABC-type transporter related to the transport of oligo- and dipeptide fragments. Unlike the putative phage sequences in the DSM16790 genome, which appear to be related to viral propagation, this series of sequences introduce putative ecophysiologically relevant genes to J07HQX50 and its related sequences.

1. Rinke C, Schwientek P, Sczyrba A, Ivanova NN, Anderson IJ, Cheng J-F, Darling A, Malfatti S, Swan BK, Gies EA, Dodsworth JA, Hedlund BP, Tsiamis G, Sievert SM, Liu W-T, Eisen JA, Hallam SJ, Kyrpides NC, Stepanauskas R, Rubin EM, Hugenholtz P, Woyke T: **Insights into the phylogeny and coding potential of microbial dark matter**. *Nature* 2013, **499**:431–437.

2. Markowitz VM, Korzeniewski F, Palaniappan K, Szeto E, Werner G, Padki A, Zhao X, Dubchak I, Hugenholtz P, Anderson I, Lykidis A, Mavromatis K, Ivanova N, Kyrpides NC: **The integrated microbial genomes (IMG) system.** *Nucleic Acids Res* 2006, **34**(Database issue):D344–8.

3. Oh D, Porter K, Russ B, Burns D, Dyall-Smith M: **Diversity of Haloquadratum and other haloarchaea in three, geographically distant, Australian saltern crystallizer ponds**. *Extremophiles* 2009, **14**:161–169.

4. Anderson I, Scheuner C, Göker M, Mavromatis K, Hooper SD, Porat I, Klenk H-P, Ivanova N, Kyrpides N: **Novel Insights into the Diversity of Catabolic Metabolism from Ten Haloarchaeal Genomes**. *PLoS ONE* 2011, **6**:e20237.

5. Podell S, Ugalde JA, Narasingarao P, Banfield JF, Heidelberg KB, Allen EE: **Assembly-Driven Community Genomics of a Hypersaline Microbial Ecosystem**. *PLoS ONE* 2013, **8**:e61692.

6. Legault BA, Lopez-Lopez A, Alba-Casado JC, Doolittle WF, Bolhuis H, Rodriguez-Valera F, Papke RT: **Environmental genomics of "Haloquadratum walsbyi" in a saltern crystallizer indicates a large pool of accessory genes in an otherwise coherent species.** *BMC Genomics* 2006, **7**:171.

7. Podell S, Emerson JB, Jones CM, Ugalde JA, Welch S, Heidelberg KB, Banfield JF, Allen EE: **Seasonal fluctuations in ionic concentrations drive microbial succession in a hypersaline lake community**. 2013:1–12.

8. Dyall-Smith ML, Pfeiffer F, Klee K, Palm P, Gross K, Schuster SC, Rampp M, Oesterhelt D: **Haloquadratum walsbyi : Limited Diversity in a Global Pond**. *PLoS ONE* 2011, **6**:e20968.

9. Cuadros-Orellana S, Martin-Cuadrado A-B, Legault B, D'Auria G, Zhaxybayeva O, Papke RT, Rodriguez-Valera F: **Genomic plasticity in prokaryotes: the case of the square haloarchaeon**. *ISME J* 2007, **1**:235–245.

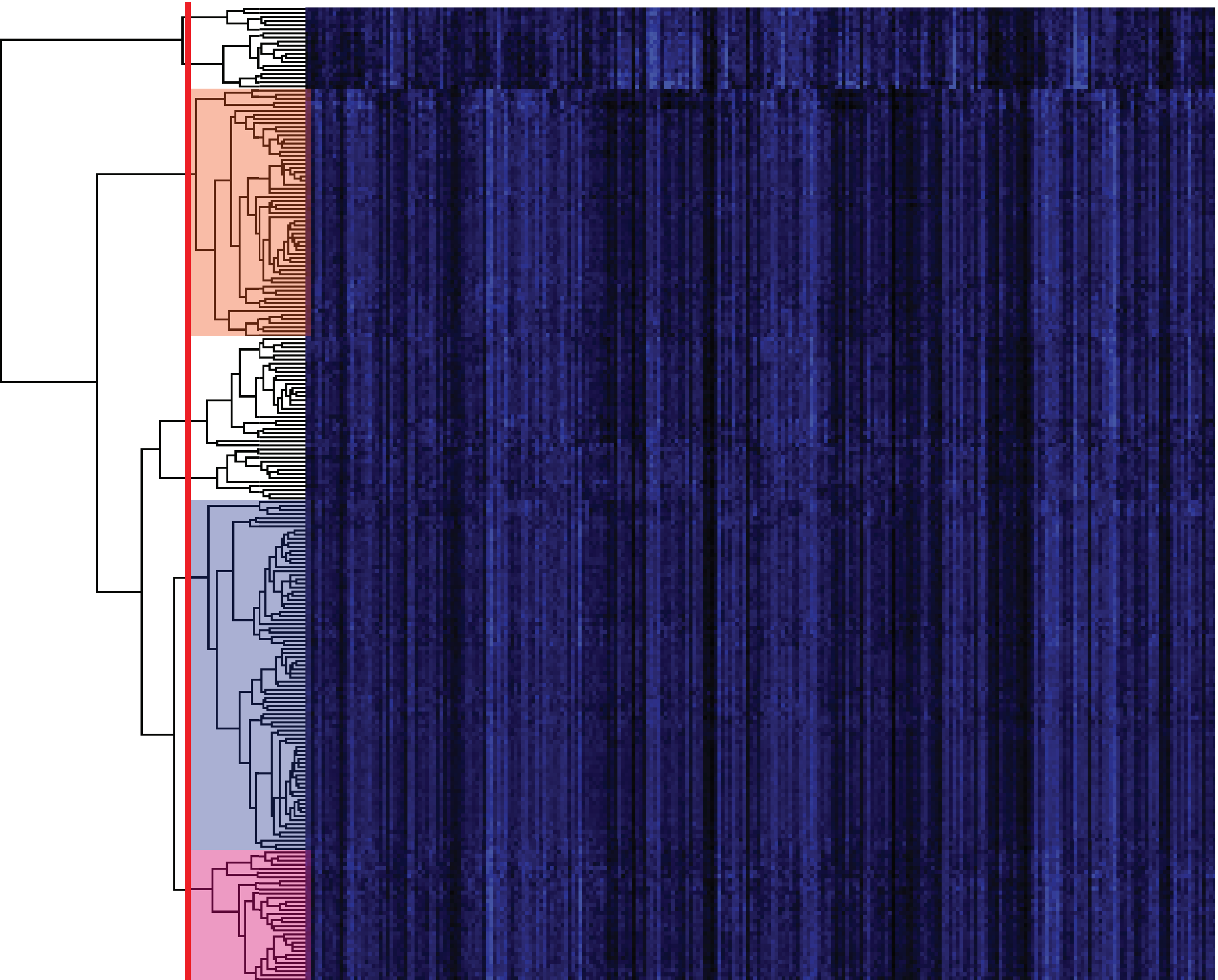

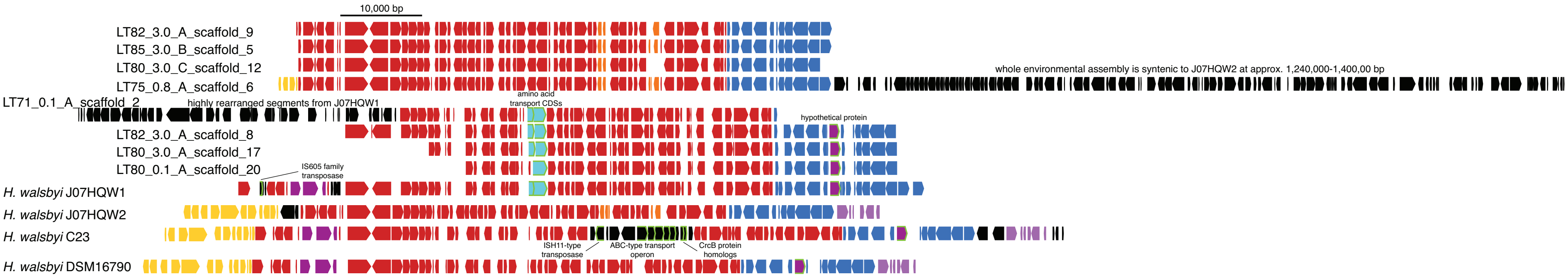

10,000 bp
LT82_3.0_A_scaffold_9
LT85_3.0_B_scaffold_5
LT80_3.0_C_scaffold_12
LT75_0.8_A_scaffold_6
whole environmental assembly is syntenic to J07HQW2 at approx. 1,240,000-1,400,00 bp
LT71_0.1_A_scaffold_2
highly rearranged segments from J07HQW1
amino acid
transport CDSs
hypothetical protein
LT82_3.0_A_scaffold_8
LT80_3.0_A_scaffold_17
LT80_0.1_A_scaffold_20
IS605 family
transposase
H. walsbyi J07HQW1
H. walsbyi J07HQW2
H. walsbyi C23
ISH11-type
transposase
ABC-type transport
operon
CrcB protein
homologs
H. walsbyi DSM16790

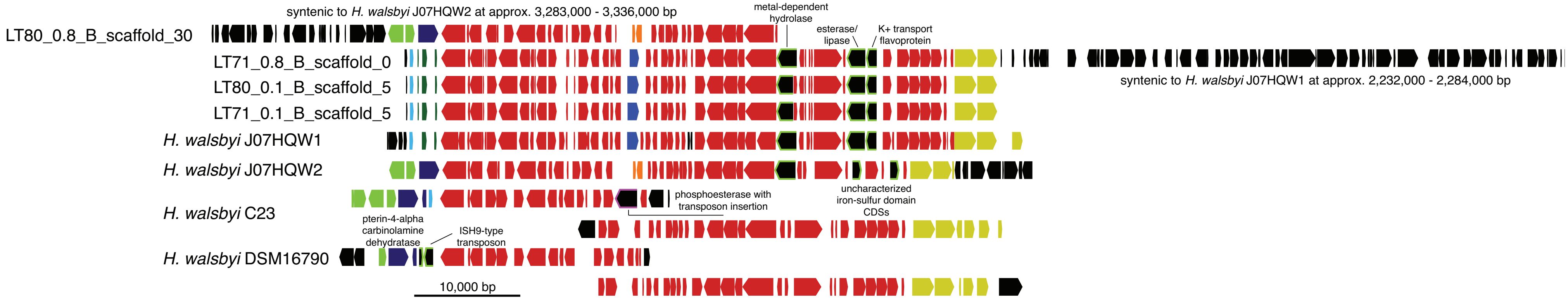

syntenic to *H. walsbyi* J07HQW2 at approx. 3,283,000 - 3,336,000 bp
metal-dependent hydrolase
esterase/ lipase
K+ transport flavoprotein
LT80_0.8_B_scaffold_30
LT71_0.8_B_scaffold_0
LT80_0.1_B_scaffold_5
LT71_0.1_B_scaffold_5
syntenic to *H. walsbyi* J07HQW1 at approx. 2,232,000 - 2,284,000 bp
H. walsbyi J07HQW1
H. walsbyi J07HQW2
H. walsbyi C23
phosphoesterase with transposon insertion
uncharacterized iron-sulfur domain CDSs
pterin-4-alpha carbinolamine dehydratase
ISH9-type transposon
H. walsbyi DSM16790
10,000 bp

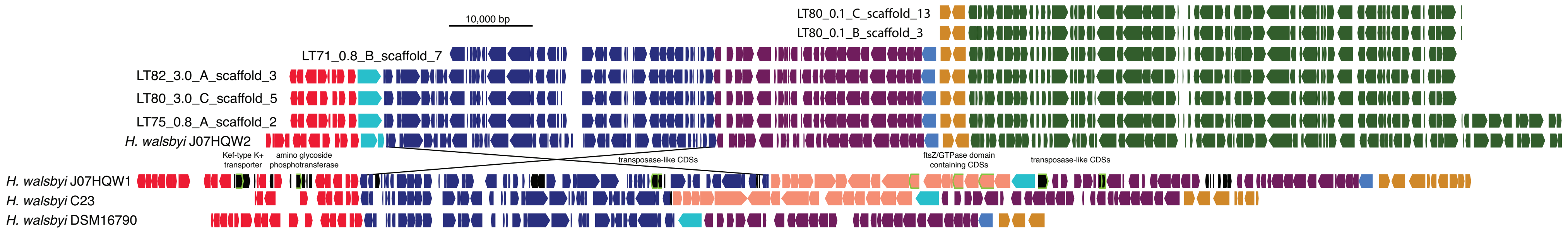

10,000 bp
LT80_0.1_C_scaffold_13
LT80_0.1_B_scaffold_3
LT71_0.8_B_scaffold_7
LT82_3.0_A_scaffold_3
LT80_3.0_C_scaffold_5
LT75_0.8_A_scaffold_2
H. walsbyi J07HQW2
Kef-type K+ transporter
amino glycoside phosphotransferase
transposase-like CDSs
ftsZ/GTPase domain containing CDSs
transposase-like CDSs
H. walsbyi J07HQW1
H. walsbyi C23
H. walsbyi DSM16790

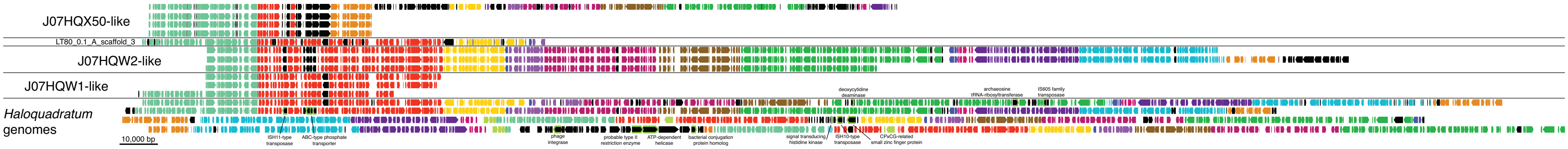
J07HQX50-like
LT80_0.1_A_scaffold_3
J07HQW2-like
J07HQW1-like
Haloquadratum genomes
deoxycytidine deaminase
archaeosine tRNA-ribosyltransferase
IS605 family transposase
10,000 bp
ISH11-type transposase
ABC-type phosphate transporter
phage integrase
probable type II restriction enzyme
ATP-dependent helicase
bacterial conjugation protein homolog
signal transducing histidine kinase
ISH10-type transposase
CPxCG-related small zinc finger protein

Supplemental Table S1 - Organism names and publication sources, where available, for the Halobacteriaceae genomes used to construct a searchable database for the first round of assembly putative open reading frames.

Organisms Name	Designation	Reference
Haloquadratum walsbyi DSM16790	*Halobacteriaceae*	Bolhuis et al 2006 [21]
Haloquadratum walsbyi C23	*Halobacteriaceae*	Dyall-Smith et al 2011 [23]
Haloquadratum walsbyi J07HQW1	*Halobacteriaceae*	Podell et al 2013 [8]
Haloquadratum walsbyi J07HQW2	*Halobacteriaceae*	Podell et al 2013 [8]
Haloquadratum sp. J07HQX50	*Halobacteriaceae*	Podell et al 2013 [8]
Halorubrum lipolyticum DSM 21995	*Halobacteriaceae*	[unpublished]
Halorubrum ezzemoulense DSM 17463	*Halobacteriaceae*	[unpublished]
Halorubrum lacusprofundi ATCC 49239	*Halobacteriaceae*	[unpublished]
Halorhabdus utahensis DSM 12940	*Halobacteriaceae*	Anderson et al 2009
Halorhabdus tiamatea SARL4B	*Halobacteriaceae*	Antunes et al 2011
Halonotius sp. J07ABHN4	*Halobacteriaceae*	Podell et al 2013 [8]
uncultured halophilic archaeon J07HR59	*Halobacteriaceae*	Podell et al 2013 [8]
Candidatus Haloredivivus sp. G17	*Halobacteriaceae*	[unpublished]
Natronorubrum tibetense DSM 13204	*Halobacteriaceae*	[unpublished]
Natronorubrum tibetense GA33	*Halobacteriaceae*	[unpublished]
Halalkalicoccus jeotgali B3	*Halobacteriaceae*	[unpublished]
uncultured halophilic archaeon J07ABHX67	*Nanohaloarchaea*	Podell et al 2013 [8]
uncultured archaeon J07ABHN6	*Nanohaloarchaea*	Podell et al 2013 [8]

**Supplemental Table S2** - Results from each round of sequencing, in detail.										Comparison between Assembly 2 and Assembly 3 Bin			
Sample Name and Filter Size	Assembly Round	No. of Reads	Bin ID	No. of contigs	N50	Max. length	Mean	Total Length	N80	N50	Max Length	Mean	Total Length
LT71 0.1	1	12845524		66991	1456	289500	1034	69290544	565				
LT71 0.1	2	861506		402	28076	145520	11305	4544755	10353				
LT71 0.1	3	576248	A	272	33837	90462	10442	2840477	11744	1.20519305	0.62164651	0.9236621	
LT71 0.1	3	397890	B	168	35294	90294	11351	1907117	14754	1.2570879	0.62049203	1.004069	
LT71 0.1	3	215820	C	84	35137	175956	17522	1471879	15718	1.25149594	1.20915338	1.54993366	1.36849467
LT71 0.8	1	4357176		17094	3800	171759	1369	23409884	660				
LT71 0.8	2	850652		416	32463	162931	13795	5738744	14130				
LT71 0.8	3	454182	A	361	39086	101513	7741	2794529	12066	1.20401688	0.62304288	0.56114534	
LT71 0.8	3	308156	B	173	39163	110156	10853	1877675	13868	1.20638881	0.67608988	0.78673432	
LT71 0.8	3	149734	C	104	35968	171649	13907	1446360	14437	1.10796907	1.05350731	1.00811888	1.06618521
LT75 0.8	1	13475176		52649	1517	265014	1003	52844405	508				
LT75 0.8	2	2905226		777	52675	265413	11248	8740423	16220				
LT75 0.8	3	1523542	A	348	51954	298502	11095	3861346	19599	0.98631229	1.12466985	0.98639758	
LT75 0.8	3	645706	B	211	47367	195592	10177	2147556	14235	0.89923113	0.73693451	0.90478307	
LT75 0.8	3	455960	C	105	97580	171475	15744	1653216	22035	1.85249169	0.64606858	1.3997155	0.87663011
LT80 0.1	1	20609138		78801	1214	268029	918	72339415	500				
LT80 0.1	2	2121606		533	50031	341847	15691	8363626	19255				
LT80 0.1	3	1170162	A	290	52155	130393	13390	3883156	22641	1.04245368	0.38143672	0.85335543	
LT80 0.1	3	734560	B	275	31814	205854	11684	3213356	13402	0.63588575	0.60218168	0.74463068	
LT80 0.1	3	679022	C	220	77612	253956	13794	3034693	18649	1.55127821	0.74289375	0.87910267	1.21134123
LT80 3.0	1	9014586		21068	2785	264822	1291	27200784	630				
LT80 3.0	2	2933418		643	57975	247354	13672	8791253	22770				
LT80 3.0	3	1486186	A	379	58466	192057	10359	3926165	23584	1.00846917	0.7764459	0.75767993	
LT80 3.0	3	1104266	B	284	63095	260635	11498	3265581	19417	1.08831393	1.05369228	0.84098888	
LT80 3.0	3	909546	C	219	89355	275698	14005	3067297	24789	1.54126779	1.11458881	1.02435635	1.16696027
LT82 3.0	1	15358950		42784	2062	264360	1121	47976572	564				
LT82 3.0	2	4003128		1027	43109	341927	8746	8982445	13291				
LT82 3.0	3	2156080	A	449	46949	264322	9362	4203897	18765	1.08907653	0.77303635	1.0704322	
LT82 3.0	3	941296	B	99	128480	195964	24933	2468437	60387	2.98035213	0.57311648	2.85078893	0.74281935
LT85 0.1	1	52520328		74812	695	166620	667	49971908	391				
LT85 0.1	2	5741106		352	28388	167894	14483	5098345	14787				
LT85 0.1	3	3557136	A	314	24854	85013	10149	3186830	14096	0.87551078	0.50634924	0.70075261	
LT85 0.1	3	3185748	B	219	44383	127230	12925	2830648	13939	1.5634423	0.75779956	0.8924256	1.18028066
LT85 3.0	1	7058560		15463	2137	265785	1186	18346377	598				
LT85 3.0	2	2659294		760	28429	229271	7197	5469932	11952				
LT85 3.0	3	1704652	A	551	28401	85607	6064	3341563	10628	0.99901509	0.37338782	0.84257329	
LT85 3.0	3	1536758	B	391	42026	232572	7546	2950727	13921	1.47827922	1.01439781	1.04849243	1.15034154

Supplemental Table S3 - Variant ABC-type transporter subunits (< 80% amino acid identity) for each of the *Haloquadratum* genomes and the environmental putative CDSs.

Organism Name	Putative Substrate	ABC-type Transporter Subunit	No. of Variants	Gene Locus IDs
H. walsbyi C23	Copper	Permease	2	Hqrw_4112, Hqrw_1278
H. walsbyi C23	Zinc	Substrate-binding	1	Hqrw_2414
H. walsbyi C23	Di-/Oligopeptide/Nickel	Substrate-binding	1	Hqrw_2776
H. walsbyi C23	No assigned substrate	ATPase	2	Hqrw_3144, Hqrw_3145
H. walsbyi C23	No assigned substrate	Permease	2	Hqrw_3142, Hqrw_3143
H. walsbyi C23	No assigned substrate	Substrate-binding	1	Hqrw_3141
H. walsbyi C23	Branched-chain amino acids	Permease	1	Hqrw_3180
H. walsbyi C23	Urea/short-chain amides	Substrate-binding	1	Hqrw_4030
H. walsbyi C23	Multidrug/lipids	ATPase	1	Hqrw_3345
H. walsbyi DSM16790	Branched-chain amino acids	Substrate-binding	1	HQ2192A
H. walsbyi DSM16790	Branched-chain amino acids	ATPase	3	HQ2193A, HQ2194A, HQ2195A
H. walsbyi DSM16790	Branched-chain amino acids	Permease	2	HQ2196A, HQ2197A
H. walsbyi DSM16790	Lipoprotein	Permease	2	HQ3476A, HQ3477A
H. walsbyi DSM16790	Lipoprotein	ATPase	1	HQ3478A
H. walsbyi DSM16790	Multidrug/lipids	ATPase	1	HQ3533A
H. walsbyi J07HWQ1	Antimicrobial peptide	ATPase	1	J07HWQ1_00013
H. walsbyi J07HWQ1	Antimicrobial peptide	Permease	2	J07HWQ1_00014, J07HWQ1_00042
H. walsbyi J07HWQ1	Unknown	Hypothetical	2	J07HWQ1_00367, J07HWQ1_00669
H. walsbyi J07HWQ1	Multidrug	ATPase	1	J07HWQ1_00516
H. walsbyi J07HWQ1	Cobalamin/Fe3+-siderophores	ATPase	1	J07HWQ1_00954
H. walsbyi J07HWQ1	Fe3+-hydroxamate	Substrate-binding	1	J07HWQ1_00956
H. walsbyi J07HWQ1	Spermidine/Putrescine	ATPase	2	J07HWQ1_01722, J07HWQ1_03537
H. walsbyi J07HWQ1	Nuceloside	Permease	1	J07HWQ1_01905
H. walsbyi J07HWQ2	Spermidine/Putrescine	Substrate-binding	1	J07HWQ2_00275
H. walsbyi J07HWQ2	Spermidine/Putrescine	ATPase	3	J07HWQ2_00810, J07HWQ2_02054, J07HWQ2_03674
H. walsbyi J07HWQ2	Spermidine/Putrescine	Permease	1	J07HWQ2_00807
H. walsbyi J07HWQ2	Nitrate/Sulfonate/Bicarbonate	ATPase	2	J07HWQ2_00783, J07HWQ2_00875
H. walsbyi J07HWQ2	Nitrate/Sulfonate/Bicarbonate	Permease	4	J07HWQ2_00781, J07HWQ2_00876, J07HWQ2_00877, J07HWQ2_03558
H. walsbyi J07HWQ2	Di-/Oligopeptide/Nickel	ATPase	3	J07HWQ2_00823, J07HWQ2_00841, J07HWQ2_02905
H. walsbyi J07HWQ2	Di-/Oligopeptide/Nickel	Permease	2	J07HWQ2_00824, J07HWQ2_00825
H. walsbyi J07HWQ2	Phosphate (PhoT family)	ATPase	2	J07HWQ2_03476, J07HWQ2_03477
H. walsbyi J07HWQ2	Phosphate (PhoT family)	Permease	1	J07HWQ2_0378
H. walsbyi J07HWQ2	Phosphate/Phosphonate	ATPase	1	J07HWQ2_01804
H. walsbyi J07HWQ2	Phosphate/Phosphonate (PhnE family)	Permease	1	J07HWQ2_01803
H. walsbyi J07HWQ2	Phosphate/Phosphonate/Phosphite	Substrate-binding	1	J07HWQ2_01805
H. walsbyi J07HWQ2	Lipoprotein	Permease	1	J07HWQ2_02156
H. walsbyi J07HWQ2	Multidrug	ATPase	2	J07HWQ2_02810, J07HWQ2_02973
H. walsbyi J07HWQ2	Cobalt (Co2+)	Permease	1	J07HWQ2_02252
H. walsbyi J07HWQ2	Branched-chain amino acids	ATPase	2	J07HWQ2_03669, J07HWQ2_03670
H. walsbyi J07HWQ2	Branched-chain amino acids	Permease	2	J07HWQ2_03665, J07HWQ2_03668
H. sp. J07HQX50*	Unknown	Hypothetical	1	J07HQXv2_01450
H. sp. J07HQX50*	Spermidine/Putrescine	Substrate-binding	1	J07HQXv2_01469
H. sp. J07HQX50*	Glycine betaine/choline-binding lipoprotein	Permease	1	J07HQXv2_02756
H. sp. J07HQX50	Spermidine/Putrescine	Substrate-binding	2	J07HQXv2_00141, J07HQXv2_02180
H. sp. J07HQX50	Spermidine/Putrescine	ATPase	5	J07HQXv2_00029, J07HQXv2_00142, J07HQXv2_01104, J07HQXv2_01468, J07HQXv2_02179
H. sp. J07HQX50	Spermidine/Putrescine	Permease	6	J07HQXv2_00143, J07HQXv2_00144, J07HQXv2_01466, J07HQXv2_01467, J07HQXv2_02181, J07HQXv2_02182
H. sp. J07HQX50	Phosphate (PhoT family)	Substrate-binding	1	J07HQXv2_00039
H. sp. J07HQX50	Phosphate (PhoT family)	ATPase	1	J07HQXv2_00042
H. sp. J07HQX50	Phosphate (PhoT family)	Permease	1	J07HQXv2_00040
H. sp. J07HQX50	Phosphate (PstA family)	Permease	1	J07HQXv2_00041
H. sp. J07HQX50	Phosphate/Phosphonate	ATPase	1	J07HQXv2_00914
H. sp. J07HQX50	Phosphate/Phosphonate (PhnE family)	Permease	3	J07HQXv2_01449, J07HQXv2_02196
H. sp. J07HQX50	Phosphate/Phosphonate/Phosphite	Substrate-binding	1	J07HQXv2_02198
H. sp. J07HQX50	Phosphate	ATPase	1	J07HQXv2_01508
H. sp. J07HQX50	Phosphate	Permease	3	J07HQXv2_01509, J07HQXv2_01510
H. sp. J07HQX50	Phosphate	Substrate-binding	1	J07HQXv2_01512
H. sp. J07HQX50	Di-/Oligopeptide	ATPase	3	J07HQXv2_00187, J07HQXv2_01439, J07HQXv2_02660
H. sp. J07HQX50	Di-/Oligopeptide/Nickel	Permease	3	J07HQXv2_01565, J07HQXv2_02659
H. sp. J07HQX50	Di-/Oligopeptide	Substrate-binding	1	J07HQXv2_02658
H. sp. J07HQX50	Sugar	ATPase	5	J07HQXv2_01051, J07HQXv2_01140, J07HQXv2_01504, J07HQXv2_01505, J07HQXv2_01506
H. sp. J07HQX50	Sugar	Permease	3	J07HQXv2_01088, J07HQXv2_01105, J07HQXv2_01422
H. sp. J07HQX50	Sugar	Substrate-binding	7	J07HQXv2_00624, J07HQXv2_01108, J07HQXv2_01138, J07HQXv2_01426, J07HQXv2_01501, J07HQXv2_02268, J07HQXv2_02570
H. sp. J07HQX50	Fe3+-hydroxamate	Substrate-binding	1	J07HQXv2_00306
H. sp. J07HQX50	Fe3+-siderophore	Permease	1	J07HQXv2_00468
H. sp. J07HQX50	Fe3+	Substrate-binding	1	J07HQXv2_01074
H. sp. J07HQX50	Fe3+	Permease	1	J07HQXv2_01075
H. sp. J07HQX50	Cobalamin/Fe3+-siderophores	ATPase	1	J07HQXv2_00467
H. sp. J07HQX50	Carbohydrate (CUT1 family)	Substrate-binding	1	J07HQXv2_01045
H. sp. J07HQX50	Carbohydrate (CUT1 family)	ATPase	5	J07HQXv2_00620, J07HQXv2_01086, J07HQXv2_01421, J07HQXv2_01528, J07HQXv2_02571
H. sp. J07HQX50	Carbohydrate (CUT1 family)	Permease	6	J07HQXv2_01046, J07HQXv2_01047, J07HQXv2_01087, J07HQXv2_01106, J07HQXv2_01502, J07HQXv2_01503
H. sp. J07HQX50	Cobalt (Co2+) (CbiQ family)	Permease	2	J07HQXv2_02186, J07HQXv2_02794
H. sp. J07HQX50	Cobalt (Co2+)	ATPase	1	J07HQXv2_02793
H. sp. J07HQX50	Branched-chain amino acids	Permease	4	J07HQXv2_01058, J07HQXv2_01059, J07HQXv2_02231, J07HQXv2_02232
H. sp. J07HQX50	Branched-chain amino acids	Substrate-binding	1	J07HQXv2_01060
H. sp. J07HQX50	Amino acid/amide (HAAT family)	Substrate-binding	1	J07HQXv2_01527
H. sp. J07HQX50	Amino acid/amide (HAAT family)	ATPase	1	J07HQXv2_01057
H. sp. J07HQX50	Amino acid/amide (HAAT family)	Permease	2	J07HQXv2_01525, J07HQXv2_01526
H. sp. J07HQX50	Multidrug	ATPase	5	J07HQXv2_00628, J07HQXv2_00763, J07HQXv2_00865, J07HQXv2_01173, J07HQXv2_02576
H. sp. J07HQX50	Monosaccaride (CUT2 family)	ATPase	1	J07HQXv2_02266
H. sp. J07HQX50	Monosaccaride (CUT2 family)	Permease	1	J07HQXv2_01139
H. sp. J07HQX50	Ribose/xylose/arabinose/galactoside	Permease	1	J07HQXv2_02267
Environmental subunit*	Urea	Substrate-binding	1	
Environmental subunit*	Nitrate	Substrate-binding	1	
Environmental subunit*	Unknown	ATPase	2	
Environmental subunit*	Oligopeptide	Nonfunctional	1	
Environmental subunit	Sugar	Permease	10	
Environmental subunit	Spermidine/Putrescine	Permease	1	
Environmental subunit	Spermidine/Putrescine	ATPase	3	
Environmental subunit	Phosphate/Phosphonate	Substrate-binding	5	
Environmental subunit	Phosphate/Phosphonate	Permease	5	
Environmental subunit	Phosphate	ATPase	10	
Environmental subunit	Oligopepdite (OppF family)	ATPase	7	
Environmental subunit	Oligopepdite (OppD family)	ATPase	3	
Environmental subunit	Nitrate/Sulfonate/Bicarbonate	Substrate-binding	2	
Environmental subunit	Nitrate/Sulfonate/Bicarbonate	Permease	9	
Environmental subunit	Nitrate	Substrate-binding	4	
Environmental subunit	Gylcerol-3-phosphate	Permease	2	
Environmental subunit	Gylcerol-3-phosphate	Substrate-binding	1	
Environmental subunit	Gylcerol-3-phosphate	ATPase	2	
Environmental subunit	Dihydroxyacetone	Permease	1	
Environmental subunit	Cobalt (Co2+)	Permease	2	
Environmental subunit	Branched-chain amino acids	ATPase	8	
Environmental subunit	Cobalamin (BtuF family)	Substrate-binding	6	
Environmental subunit	Unknown	Permease	7	
Environmental subunit	Unknown	ATPase	8	

*Represent putative CDS without orthologs in the *Haloquadratum* genomes

**Supplmental Table S4** - Recruitment statistics of the samples with multiple filter fractions				
Sample	No. of Sequences in Library	No. of J07HQW1 Recruited Sequences (% Total)	No. of J07HQW2 Recruited Sequences (% Total)	No. of J07HQX50 Recruited Sequences (% Total)
LT71 0.1 um	12,845,524	847,324 (6.6%)	664,844 (5.2%)	185,519 (1.4%)
LT71 0.8 um	4,357,176	659,273 (15.1%)	483,323 (11.1%)	158,476 (3.6%)
LT80 0.1 um	20,609,138	1,330,681 (6.5%)	890,207 (4.3%)	577,845 (2.8%)
LT80 3.0 um	9,014,584	1,684,250 (18.7%)	1,242,998 (13.8%)	661,228 (7.3%)
LT85 0.1 um	52,520,328	4,068,426 (7.7%)	4,117,218 (7.8%)	619,120 (1.1%)
LT85 3.0 um	7,058,560	2,024,765 (28.7%)	2,119,851 (30.0%)	277,659 (3.9%)
